# A health promotion intervention for vulnerable schools (BeE-school): a cluster-randomized trial: Rafaela Rosário

**DOI:** 10.1093/eurpub/ckac131.339

**Published:** 2022-10-25

**Authors:** R Rosário, B Pereira, P Novais, H Antunes, MJ Silva, C Augusto

**Affiliations:** 1 School of Nursing, University of Minho, Braga, Portugal; 2 Health Sciences Research Unit: Nursing, Nursing School of Coimbra, Coimbra, Portugal; 3 Research Centre in Child Studies, Institute of Education, University of Minho, Braga, Portugal; 4 Algoritmi Center, Department of Informatics, University of Minho, Braga, Portugal; 5 Life and Health Sciences Research Institute, University of Minho, Braga, Portugal; 6 School of Medicine, University of Minho, Braga, Portugal

## Abstract

**Background:**

Noncommunicable diseases (NCDs) share key determinants like unhealthy diet, unhealthy 24h- movement behaviour (sleep, sedentary behaviour and physical activity), leading to other risks including overweight, obesity and raised blood pressure. Although the manifest of the NCDs in childhood is rare, risk behaviours, obesity, and raised blood pressure that accelerates their development begin during childhood. The current study aims to analyze the effectiveness of the intervention program based on the promotion of health literacy and lifestyles, on children’s health literacy, lifestyles (e.g. dietary intake, 24hmovement behaviour) and overweight and obesity.

**Methods:**

478 children (6 schools) aged 6-12years old will participate in this cluster-randomized trial, having schools as the unit of randomization, assigned into intervention (239-3schools) and the control arm (239-3schools). This project is currently performing social listening (online and offline) and stakeholders’ involvement. Data collection includes sociodemographics, health literacy and infodemic resilience, dietary intake and children’s 24-h movement behaviour (e.g. accelerometry), anthropometry (e.g. weight, height and waist circumference) and blood pressure. It will occur at baseline and after the intervention (follow-up, 6 months after the beginning of the intervention).

**Results:**

Expected outputs and outcomes include the 1-creation of a model for characterizing NCDs and health topics based on artificial intelligence techniques (e.g. deep learning, social network analysis methods).2- improved health literacy and infodemic resilience of children, families and teachers.3- enhanced children’s lifestyles. 4- reduce NCDs’ physical risk factors (e.g. overweight, raised blood pressure).

**Conclusions:**

A feasible intervention program for school-aged children with vulnerabilities enhances tailored policies about health promotion and NCDs’ prevention, respecting the contextś singularities.

**Key messages:**

• Health promotion and NCDs prevention are crucial for the well-being of our societies.

• Feasible intervention programs advocates for evidence-based policies that respect local singularities.

